# Urinary Metabolites Altered during the Third Trimester in Pregnancies Complicated by Gestational Diabetes Mellitus: Relationship with Potential Upcoming Metabolic Disorders

**DOI:** 10.3390/ijms20051186

**Published:** 2019-03-08

**Authors:** Yamilé López-Hernández, Ana Sofía Herrera-Van Oostdam, Juan Carlos Toro-Ortiz, Jesús Adrián López, Mariana Salgado-Bustamante, Michael Murgu, Lourdes Mariela Torres-Torres

**Affiliations:** 1 Metabolomics and Proteomics Laboratory, CONACyT-Universidad Autónoma de Zacatecas, 98066 Zacatecas, Mexico; 2 Biochemistry Department, Universidad Autónoma de San Luis Potosí, 7800 San Luis Potosí, Mexico; chromosomesxx.svo@gmail.com (A.S.H.-V.O.); mariana.salgado@uaslp.mx (M.S.-B.); 3 Gynecology and Obstetrics Division, Hospital Central “Dr. Ignacio Morones Prieto”,7800 San Luis Potosí, Mexico; jcarlostoro@yahoo.com.mx (J.C.T.-O.); mariela.torres161291@gmail.com (L.M.T.-T.); 4 MicroRNAs Laboratory, Unidad Académica de Ciencias Biológicas, Universidad Autónoma de Zacatecas, 98066 Zacatecas, Mexico; jalopez@uaz.edu.mx; 5 Waters Technologies of Brazil, 06400 Barueri, Brazil; michael_murgu@waters.com

**Keywords:** gestational diabetes mellitus, metabolomics, urine, tryptophan, mass spectrometry

## Abstract

Gestational diabetes mellitus (GDM) is a disorder in pregnancy with highest impact in the future life of both mother and newborn. Increasing incidence, economic impact, and potential for severe GDM-related pregnancy complications are some factors that have motivated the deep study of physiopathology, risk factors for developing GDM, and potential biomarkers for its diagnosis. In the present pilot study, we analyzed the urinary metabolome profile of GDM patients in the 3rd trimester of pregnancy, when GDM is already established and the patients are under dietary and pharmacological control. An untargeted metabolomics method based on liquid chromatography–mass spectrometry analysis was developed to identify differentially expressed metabolites in the GDM group. We identified 14 metabolites that are significantly upregulated in the urine of GDM patients, and, more importantly, we identified those related with the steroid hormone biosynthesis and tryptophan (TRP) metabolism pathways, which are associated with GDM pathophysiology. Thus, these metabolites could be screened as potential prognostic biomarkers of type two diabetes mellitus, coronary artery disease and chronic renal failure in future follow-up studies with GDM patients.

## 1. Introduction

Gestational diabetes mellitus (GDM) is the most common complication of pregnancy worldwide and is defined as hyperglycemia that is recognized for the first time during pregnancy [1]. The disease is usually diagnosed in the second or third trimester of pregnancy in patients with no history of diabetes prior to gestation [2].

In Mexico, the prevalence of GDM ranges from 8.7–17.7%. Women in Mexico are a high-risk group for developing GDM, which increases in incidence in older, overweight and obese women (body mass index > 30 kg/m^2^), or those with a family history of type two diabetes (T2D) [3,4].

Hyperinsulinism with increased peripheral insulin resistance (IR) is a characteristic of GDM. Hyperglycemia during pregnancy promotes epigenetic changes to the fetus and is associated with increased risk for chronic diseases during adult life [5]. Maternal hyperglycemia results in exaggerated fetal anabolism, growth of fetal adipose tissue, and bone and muscle tissue leading to macrosomia. The newborns are prone to neonatal hypoglycemia, hyperbilirubinemia, hypocalcemia, respiratory distress syndrome, and polycythemia [6].

Women with GDM are, on average, seven times more likely to develop T2D. Approximately 50% of mothers with GDM will develop T2D within ten years—along with cardiovascular morbidity, metabolic syndrome, and renal complications [7,8]. However, the metabolic mechanisms underlying this pathophysiology remain poorly understood. Metabolomics studies have confirmed that highest circulating concentrations of different metabolites in patients with T2D and GDM are associated with IR and pancreatic β-cell dysfunction [9]. A range of untargeted and targeted metabolomics methodologies have been developed to characterize the metabolome. Amino acids and its derivatives—organic acids, lipids, and fatty acids—are some dysregulated metabolites identified in studies conducted principally with maternal serum or plasma [10]. Only a few studies have investigated the excretion profile of maternal urine [11,12,13,14,15,16]. Urine is very useful for clinical applications because it is available in large quantities, can be collected in a non-invasive manner, and sample treatment is relatively simple. However, the biological interpretation of the urine metabolome remains challenging due to the effect of physiological factors or hydration status.

While the search for biomarkers capable of predicting GDM early in pregnancy (first and second trimesters) has been the main goal of metabolomics studies conducted in pregnant women, it is also very important to investigate the metabolic alterations in late pregnancy, when GDM has been established. The identification of dysregulated circulating metabolites in GDM patients—metabolites which have also been found to be dysregulated in patients with diabetes, renal failure, cardiovascular complications, and hypertensive disorders—may reflect unmanaged GDM or ineffective response to treatment and dietary control, leading to the aforementioned diseases. Recently, dysregulated tryptophan (TRP) and purine metabolism have been described as the major pathophysiology of GDM [16]. Besides, chronic kidney disease secondary to T2D is also associated with accumulation of toxic TRP metabolites due to both inflammation and impaired kidney function [17]. Dysregulation of TRP-kynurenine (KYN) and KYN-nicotinamide adenine dinucleotide (NAD) metabolic pathways has been postulated as one of the mechanisms of IR [18]. Recently, branched-chain amino acids (BCAAs) and the valine metabolite 3-hydroxybutyrate have shown potential as biomarkers for the transition of GDM to T2D [19]. In conjunction, the study of metabolic pathways dysregulated during the course of GDM could contribute to predicting irreversible metabolic effects in the mother.

In the present study, we analyzed the urinary metabolome of Mexican GDM patients at the 3rd trimester of pregnancy, when GDM is already established and the patients are under dietary and pharmacological control, with the purpose of predicting dysregulated metabolic pathways that could be molecular links associated with negative outcomes after pregnancy.

## 2. Results

### 2.1. Clinical and Demographic Characteristics of the Groups Under Study

A total of 35 pregnant women were selected for the study from an initial cohort of 80 patients. 11 women had healthy pregnancies (control group) and 24 patients had GDM (GDM group). The clinical characteristics of the study groups are presented in Table 1. These data belong to the first prenatal control. There were no statistically significant differences between groups for maternal age, pre-gestational body mass index (BMI), and all clinical findings except glucose levels (*p* = 0.0029).

**Figure 1:**
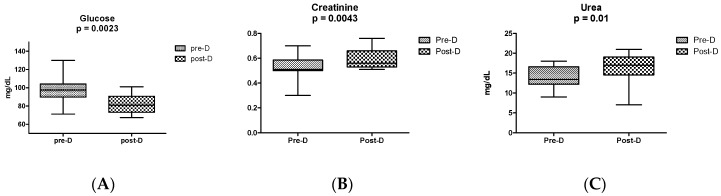
Levels of (**A**) glucose, (**B**) creatinine, and (**C**) urea measured in plasma of GDM patients before and after GDM diagnosis. Significant differences between the levels in the first prenatal visit (pre-D) and the levels at 3rd trimester (post-D) were found for the three metabolites (*p* < 0.05). Data were analyzed by a paired t-test with the software GraphPad Prism.

The urea, creatinine, and glucose results measured in the plasma of GDM patients before and after the diagnosis are shown in Figure 1. 

### 2.2. Ultra-Performance Liquid Chromatography (UPLC)-Mass Spectrometry (MS) Urinary Profiles

**Figure 2:**
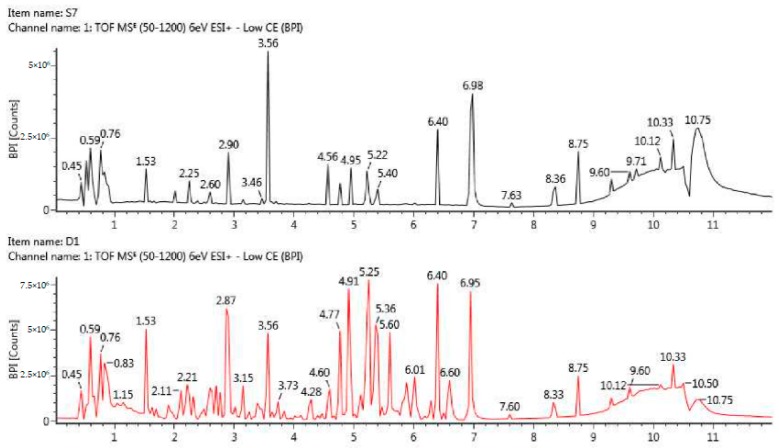
Positive ion base peak intensity chromatograms of urine from a healthy control (top) and a GDM patient (bottom) in the third trimester.

Typical urinary profiles acquired under positive ion mode are shown in Figure 2.

**Figure 3:**
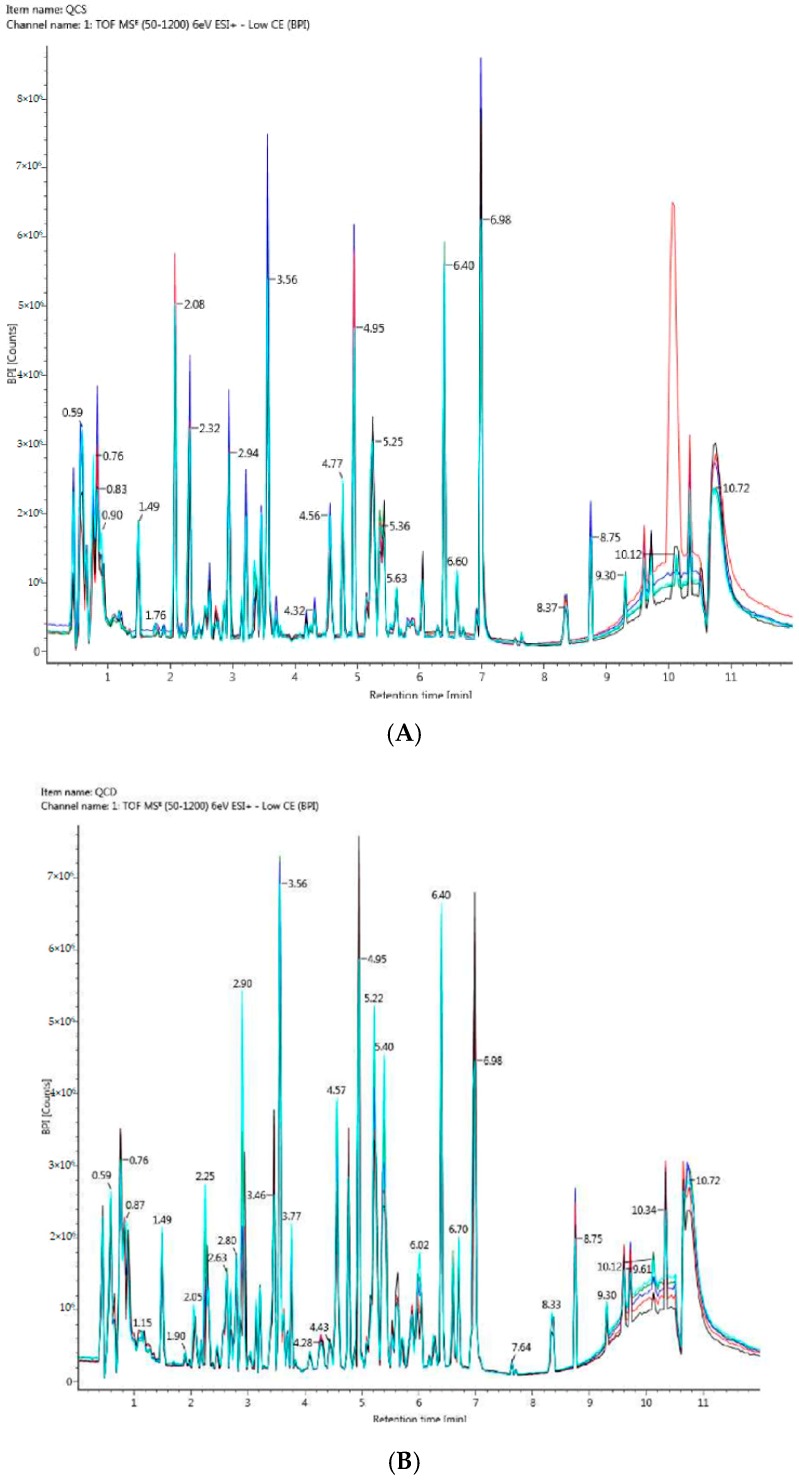
Overlaid chromatograms of quality controls (QC) showing good reproducibility for retention time, peak shape, and peak intensity. (**A**) QCS: overlaid chromatograms of five replicates of QC from the control group. (**B**) QCD: overlaid chromatograms of five replicates of QC from the GDM group.

The methodology’s reproducibility was evaluated between quality control (QC) injections, which were run before the samples, and showed good reproducibility for retention time, peak shape, and peak intensity. These were evaluated by direct comparison of overlaid chromatograms (Figure 3A,B) that showed no drifts in retention time, reflecting the stability and reproducibility of the system.

### 2.3. Metabolites Identification

A total of 4598 features were detected under the conditions employed for the pre-processing of the raw data within UNIFI 1.8.1 (Waters Corp., Milford, MA, USA) and detailed in the Material and Methods section (retention times from 0 to 10 min). Since creatinine normalization had been proven inappropriate for clinical applications [20], in the present study normalization was applied using total ion abundance, scaling the summed abundance of all compound ions in each sample to an equal value.

In the principal component analysis (PCA) score plot, the samples of the control group were only partially separated from the GDM group (variance explained R^2^Xcum = 60%). PCA was first performed to discover intrinsic treatment-related clusters within the datasets and to identify outliers (Supplementary Figure S1). 

**Figure 4:**
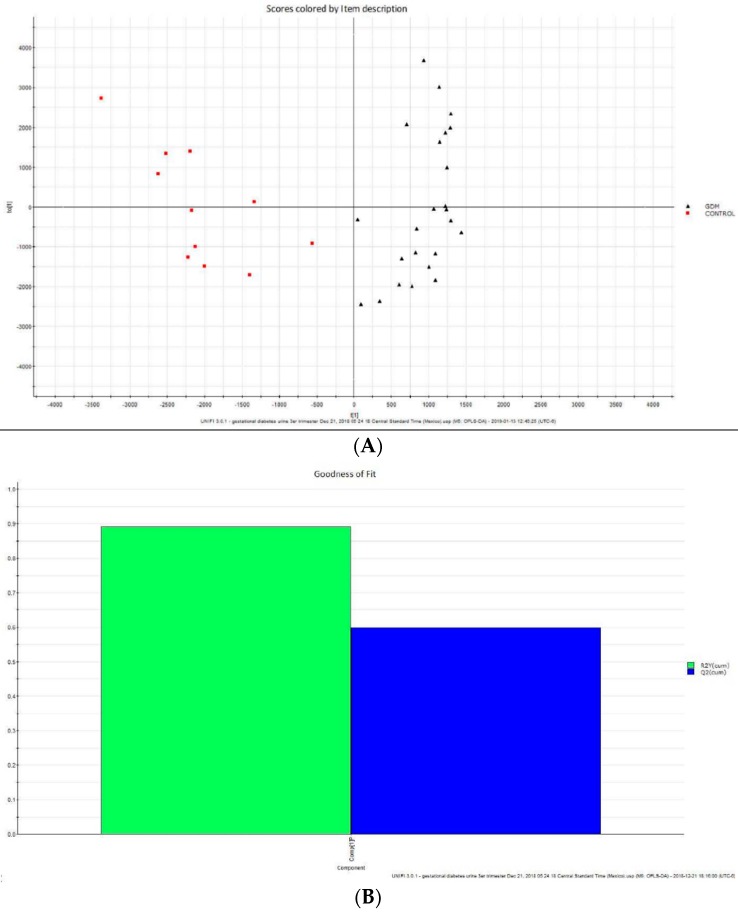

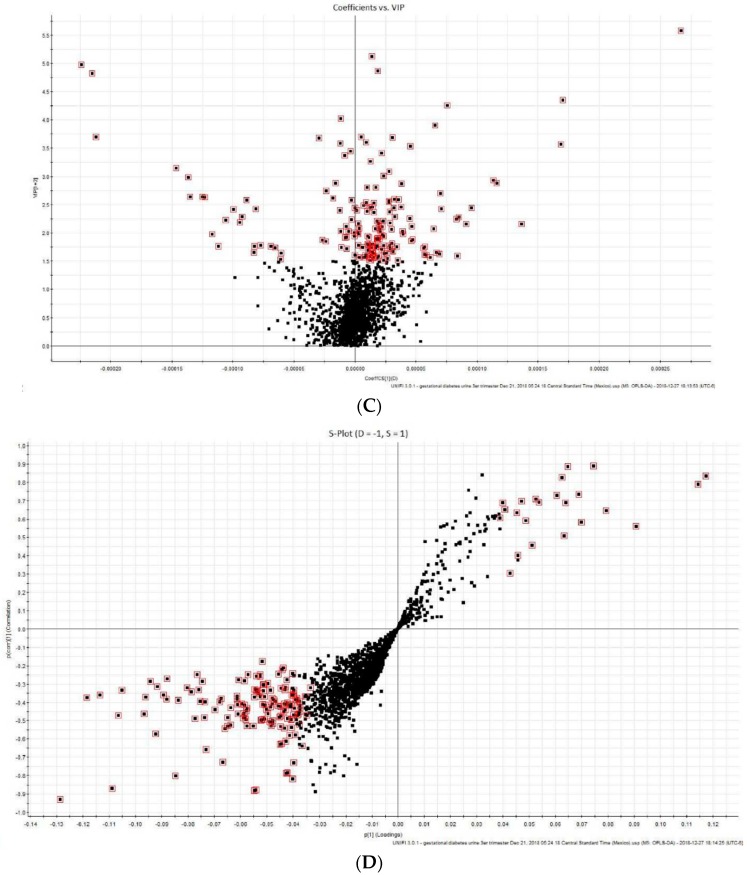
(**A**): Orthogonal partial least-squares discriminant analysis (OPLS-DA) score plots of the urine data set, acquired under positive ion mode. Visual inspection of the OPLS-DA score plot exhibited tight clusters of samples from each group. (**B**): Goodness of fit; variance explained R^2^Y(Cum): 88%, variance predicted Q^2^(Cum): 59%. (**C**): Coefficients vs. variable importance in the projection (VIP). The VIP values were also implemented to search for potential biomarkers. Only variables with VIP values higher than 1.5 were highlighted to be important for discrimination. (**D**): S-plot score plot.

Following this, partial least-squares discriminant analysis (PLS-DA) and orthogonal partial least-squares discriminant analysis (OPLS-DA) were used to improve separation among the groups and to screen for differential metabolites. The OPLS-DA score plots resulted in inter-group separation (Figure 4A). The parameters of the obtained models were satisfied with an acceptable quality of variance explained (R^2^) and variance predicted (Q^2^) and are represented in Figure 4B. Differential metabolites were selected based on the separation through the OPLS-DA loadings and variable importance projection (VIP) scores (Figure 4C). VIP represents the extracted variables’ ability to discriminate between different treatments. The variables with VIP values greater than 1.5 (VPI > 1.5) were included in the set of metabolites analyzed. The loading plots (S-Plot) identified the metabolites with significant differences in abundance between the study groups (Figure 4D). These variables were further filtered by Mann–Whitney–Wilcoxon test to determine whether a potential biomarker was statistically significant between the two groups. The metabolites with a significant difference after false discovery rate correction (*q* < 0.05) were kept and considered as the potential biomarkers.

The metabolites selected in the OPLS-DA loading S-plot were identified as described in Material and Methods. Identified metabolites with significant changes in expression after false discovery rate (FDR) correction in the GDM group are summarized in Table 2. Moreover, Supplementary Table S1 shows other identified metabolites (*p* < 0.05; *q* > 0.05). The exact measured mass/charge (*m*/*z*), mass error (mDa), retention time, and percentage of changes between groups (fold change) are detailed along with the statistical significance of each change. The selected metabolites were identified and classified according to their degree of physicochemical and/or spectral similarity to published data. Mass Spectrometry (MS^E^) data were manually inspected for the correct identification of major ions.

### 2.4. Pathway Analysis

**Figure 5:**
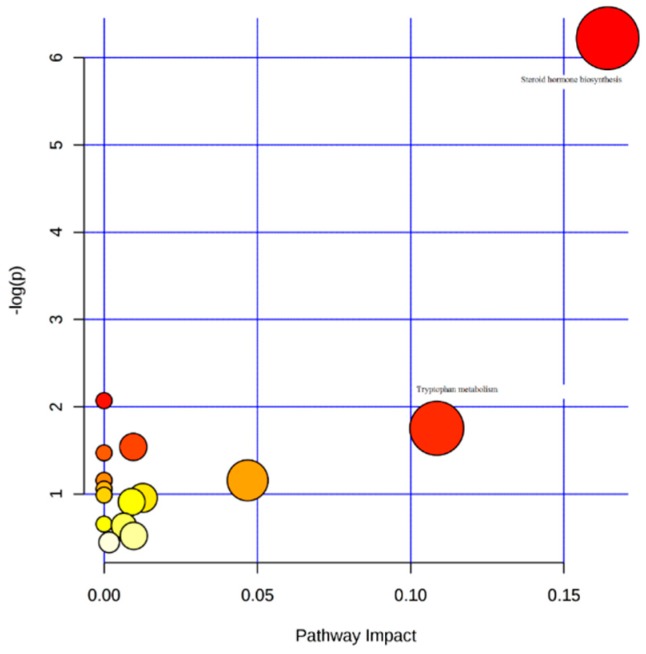
Summary of pathway analysis visualized by MetPA. Steroid hormone biosynthesis and tryptophan metabolism pathways have significant pathway impact (pathway impact > 0.1). The dots represent the pathways that were matched using pathway impact values from pathway topology analysis and p values from pathway enrichment analysis. Colors (varying from yellow to red), means the metabolites are in our data with different levels of significance for enrichment analysis. Other metabolic pathways identified are: Sphyngolipid metabolism, Phenylalanine, Tyrosine and Tryptophan metabolism, Nitrogen metabolism, Nicotinate and nicotinamide metabolism, Glycine, serine and threonine metabolism, Starch and sucrose metabolism, Pentose and glucuronate interconversions, Aminocyl t-RNA biosynthesis, Arginine and proline metabolism, and Purine metabolism.

The underlying signaling pathways and molecular networks influenced by GDM were explored and visualized by MetPA, a web application for metabolomics analysis. Identified metabolites contributing to the separation of pairwise groups were imported into MetPA. The “Homo sapiens” library was selected for the database, while hypergeometric test and relative-betweenness centrality were performed for over-representation analysis and pathway topology analysis respectively. Tryptophan metabolism and steroid hormone biosynthesis were classified as important, although other metabolic pathways related to amino acids, lipids, purine, and carbohydrate metabolism were also identified (Figure 5). 

**Figure 6:**
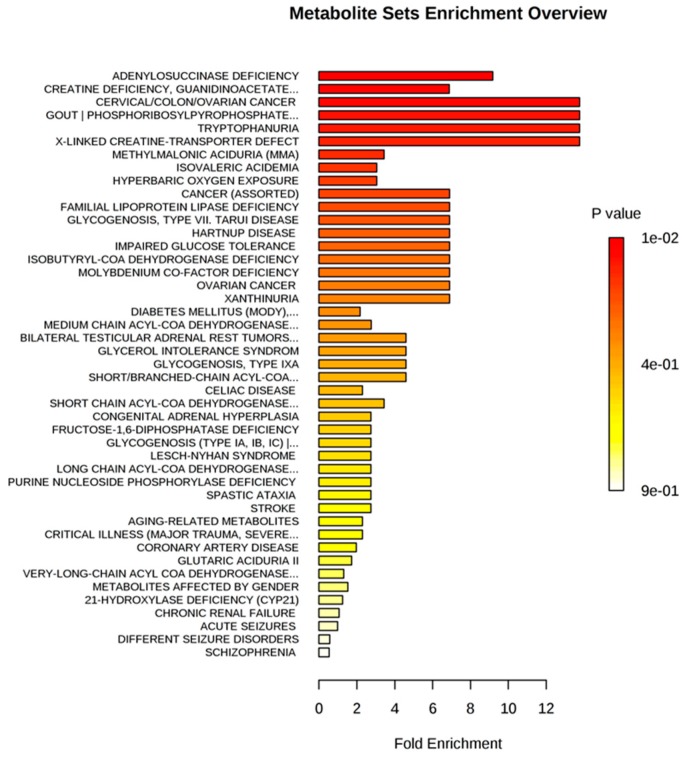
Metabolite set enrichment analysis for the prediction of disease associated metabolite sets (urine).

When investigated with the same methodology for the relationship of urinary metabolites with diseases, cervical/colon/ovarian cancer, impaired glucose tolerance, diabetes mellitus, coronary artery disease, and chronic renal failure, among others, were predicted (Figure 6). 

## 3. Discussion

In the present pilot study, we analyzed the urinary metabolomics profiles of patients with GDM in the third trimester of pregnancy. At this point, all the patients enrolled in the study were receiving dietary control and/or pharmacological support (insulin or metformin or a combination of both). Since GDM was diagnosed at 24–28 weeks of gestation, the hyperglycemia associated with this condition could have induced persistent metabolic alterations during the pregnancy. The selected participants were matched according to pre-gestational BMI and age, which, although non-significant, were higher on average in the GDM group. In both groups, consistent with epidemiologic statistical reports in the Mexican population [3], overweight and obese women were predominant. As shown in Figure 1, the evolution of the disease was monitored measuring the levels of glucose, creatinine, and urea before and after the GDM diagnosis. The levels of glucose decreased significantly at the 3rd trimester, reflecting a positive effect of treatment and dietary control (three patients received insulin treatment and 21 were treated with metformin 850 mg). However, the significant increase in the levels of serum urea and creatinine in the GDM group after diagnosis means that underlying metabolic disorders continued taking place during the pregnancy. The significantly upregulated metabolites identified in our work by the metabolomics approach belong to the following compound classes: benzopyrans, carboxylic acids and derivatives, glycerolipids, indoles and derivatives, tetrapyrroles, sphingolipids, and steroid derivatives. Different metabolites within these classes have an impact in the physiopathology of GDM and its complications.

### 3.1. The Contribution of Identified Metabolites in the Physiopathology of GDM

Since the composition of urine is significantly influenced by diet, measurement of maternal urine can be used to identify a change in dietary pattern. In our study, aspartame (known as trademark canderel), 5-carboxy-alpha-chromanol (related with the Vitamin E metabolism), and cucurbitacin c (present in fruits and cucumber) are related with dietary control in the GDM group. These substances are particularly consumed in Mexican patients under a dietary regimen, as suggested by the Mexican Food System Equivalents [21]. 

Several compounds classified as steroids and derivatives were also found upregulated in our study: 11-oxo-androsterone-glucuronide, cortolone-3-glucuronide, tetrahydroaldosterone-3-glucuronide, 5-androstene-3b,16b,17a-triol, and 21-deoxycortisol. Some of these metabolites are related to the glucuronidation process—which is used to assist in the excretion of toxic substances, drugs or other substances that cannot be used as an energy source—and have been found altered during GDM [16]. The increase in cortisol cortisol derivative levels during pregnancy is considered as the main cause of the decrease in glucose tolerance. Steroid hormones, which are elevated steadily during pregnancy, are the main hormones that influence β-cell function in early pregnancy and IR, especially in late pregnancy. Estrogen levels change during pregnancy in different states of GDM [22]. 

Steroid hormones and lipid metabolism are closely related, not only because lipids are precursors of steroid hormones, but also due to the effect on lipid metabolism during pregnancy. In our study, we identified two classes of lipids: sphingolipids and glycerolipids. Diacylglycerols (DGs; belonging to glycerolipids) are intracellular messengers that have been identified as mediators of IR [23]. Regarding sphingolipid metabolism, we detected two species differentially expressed: SM (d18:0/22:0) and Cer (d18:0/23:0). Sphingomyelins are present in animal cell membranes, and the synthesis and degradation of sphingomyelin species produce signal transduction second messengers that regulate the innate immune response at the feto-maternal interface [24]. Other authors have found pronounced elevations in several species of both saturated and unsaturated sphingomyelins in GDM amniotic fluid [25]. 

The metabolic breakdown of SM results in ceramides, which are recognized as proinflammatory lipids which are increased in T2D. Ceramide accumulation has demonstrated to be detrimental to pancreatic beta cells and may promote IR, thereby playing a direct role in the pathogenesis of T2D in both the general population and in women with previous GDM [26]. 

L-tryptophan was found upregulated in our study. Altered levels of TRP have been found in GDM patients [16] as well as in normal pregnancies [9]. TRP is metabolized via TRP-KYN and TRP-methoxyindole pathways. 

Regarding the relationship between the identified metabolites and the pharmacological treatment with metformin, in a recent work, Zucker diabetic fatty rats were treated daily for 12 weeks with metformin (200 mg/Kg), which represents a high dose when compared to the maximum human daily dose of 2000 mg/day. In this study, six metabolites were found to have significantly reverted to the normal levels after the therapy, including sphingosine [27]. However, this study was conducted in a model of non-pregnant diabetic rats. A recent report informed about the metabolic profile in women with GDM treated with metformin or insulin [28]. In this study, independently of medication, pregnancy itself had marked influences on amino acid profiles. Metformin treatment of GDM caused a greater increase in serum alanine, isoleucine, and lactate concentrations; this agrees with other previously reports. It was demonstrated that treatment with metformin is associated with increased triglyceride levels and higher atherogenic index of plasma in the third trimester in pregnant women with GDM [29]. Although measures of glucose and C-reactive protein improved with treatment with metformin and insulin, the increase in maternal plasma triglycerides—between randomization to 36 weeks—was greater in women treated with metformin [30,31]. Moreover, previous randomized control trials of lifestyle advice or metformin in obese or overweight pregnant women have reported little or no effect on standard lipid measurements [32,33].

### 3.2. Pathway Analysis: Impact on GDM Complications

The metabolic pathways considered as significant (pathway impact > 0.1) in our study were the steroid hormone biosynthesis and TRP metabolism pathways. 

In pregnancy, increase in IR occurs due to substantial steroid spectra changes. Major changes in the hypothalamic–pituitary–adrenal/–gonadal axis influence fetal growth and timing of delivery. In the same manner, counter-regulatory hormones—placental growth hormone (GH), glucocorticoid cortisol, and progesterone—progressively increase. It has been reported that gonadal steroids have also been shown to modulate pancreatic function and susceptibility to developing IR and T2D. High levels of androgens are also associated with other serious health consequences, such as high cholesterol, high blood pressure, heart disease, IR, and T2D. Moreover, IR leads to hyperinsulinemia, which is described to induce androgen production and, consequently, hyperandrogenemia directly promotes peripheral IR in women [34]. These mechanisms, when dysregulated, promote the emergence of GDM.

Regarding the TRP dysregulation, in a recent study, serum TRP level was found to be significantly higher in T2D and was positively and independently associated with risk of diabetes onset. Patients with higher TRP level tended to present with a higher degree of IR, higher triglycerides, and higher blood pressure [35]. These authors suggest that serum TRP levels increased before IR and T2D, and then depleted gradually along with the progression of T2D. The variation pattern of circulating TRP may represent the compensatory metabolic response to increased oxidative stress related to inflammation as well as the competition with branched-chain amino acids for the same trans-membrane transporter during the development of T2D.

Metabolites of the TRP-kynurenine pathway (i.e., TRP, kynurenine, kynurenic acid, quinolinic acid, 3-hydroxyanthranilic) were also associated with diabetes development in another study [36]. Other authors have reported that downstream bioactive TRP metabolites—kynurenine, kynurenic acid, and quinolinic acid—were positively and robustly correlated with the severity of kidney disease [18]. The close relationship in the kynurenine pathway between TRP, gamma-interferon, and 2-3-dioxygenase (IDO) as an immuno-modulatory mechanism has since been substantiated [36].

In the specific context of TRP alterations during pregnancy, recent work has also demonstrated similar results: changes in L-TRP in the GDM group were related to an altered serotonin metabolism [37].

TRP is in high demand during pregnancy to meet the increased protein formation for the development of the fetus, and also essential for the production of serotonin in brain, melatonin in the pineal gland, nicotinic acid in liver, etc. This has led to the end for “the tryptophan depletion concept in pregnancy” and its replacement by the “tryptophan utilization concept” [38]. High levels of TRP have also found in pregnancy disorders. A potential role of excessive levels of TRP in preeclampsia has been found, suggesting that high TRP levels can undermine T-cell suppression, resulting in pregnancy complications [39].

The finding of steroid hormones and TRP metabolism dysregulation in our study may be linked with the results obtained in the metabolite set enrichment analysis, where some of the diseases that have been associated with abnormalities in these metabolites are listed (Figure 6). These diseases have been associated with previous GDM history.

Since this study is a pilot and exploratory study, it is limited by a small sample size and the lack of an external validation cohort at the time of the study. The internal cross-validation and univariate methods employed were helpful in validating the OPLS-DA model; this intriguing initial observation, however, requires external validation. Hence, it is imperative that further longitudinal studies be conducted to replicate these results using a larger and more diverse patient cohort.

## 4. Materials and Methods 

### 4.1. Study Design and Selection of Participants

A cross-sectional study was performed between May and December 2018 to evaluate the metabolomics profile in the 3rd-trimester urine of pregnant women diagnosed with GDM. The GDM group (*n* = 24) was composed of patients who were diagnosed with GDM during the second trimester. The control group (*n* = 11) was constituted by euglycemic women. Patients who developed GDM were matched with women with normal pregnancies based on age and first prenatal body mass index (BMI). The criteria used for the diagnosis of GDM were established in accordance with the parameters established by the American College of Obstetricians and Gynecologists (ACOG) [40]. A routine oral glucose tolerance test (OGTT) was performed at 24–28 weeks’ gestation, following the World Health Organization recommendations [41]. Patients enrolled in the study received treatment immediately after the diagnosis and until the delivery. The first-line treatment for pre-gestational and GDM is diet and moderate exercise, which can control up to 70–85% of patients. The first-line pharmacological treatment for gestational diabetes mellitus is insulin, however, in this study, the use of metformin in GDM patients with 20 weeks of gestation or higher was considered as a choice of treatment: (a) when the patient refused the insulin therapy, (b) when the patient was controlled without risk for the binomial, and (c) when the patient stated her agreement with the therapy having signing an informed consent form.

Table 3 summarizes the diagnostic criteria established at Hospital Central. Patients with gestational hypertension, urinary infections, pre-existent T2D, preeclampsia, and chronic renal disease were excluded. Patients were also interviewed and tested for additional comorbidities. Neither cancer nor Polycystic ovary syndrome were reported among the patients. Clinical and demographic data were collected from the medical records for each participant at the first prenatal visit. For GDM patients, levels of glucose, urea, and creatinine were also measured in the 3rd trimester.

### 4.2. Ethical Considerations

The study was carried out in agreement with the Helsinki Declaration. Signed written informed consent was obtained from all participants prior to interviews and sample collections. The protocol was approved by the Research Ethics Committee of the Hospital Central “Dr. Ignacio Morones Prieto”, San Luis Potosi, Mexico, with Registry: CONBIOETICA-24-CEI-001-201604279. The Registry number of the protocol is 84-17 and it was approved on 19 December 2017.

### 4.3. Sample Collection and Preparation

Prenatal visits were always scheduled in the morning. Midstream urine samples were collected from each patient at the Hospital Central. The samples were centrifuged at 1200 rpm for 15 min at room temperature (RT) to eliminate cells and/or cellular debris. Then, the urine samples were again centrifuged at 3000 rpm at 4 °C, aliquoted and stored at –80 °C until use. For metabolomics analysis, urine samples were thawed on ice and vortexed. A 10-microliter aliquot of each sample was pooled to build quality controls (QC) for each group under study. A 100-microliter aliquot of each sample and QCs was diluted with liquid chromatography–mass spectrometry (LC–MS) grade water (1:1 *v*/*v*). The mixture was then centrifuged at 14,000 rpm at 4°C for 15 min. The supernatant was transferred to glass sample vials for UPLC–MS analysis.

### 4.4. UPLC- MS Method for Metabolomics Analysis

LC–MS grade acetonitrile and water was purchased from JT Baker (Brick Town, NJ, USA). High- purity formic acid (99%) was provided by Thermo Scientific (Rockford, IL, USA).

Samples were analyzed with an ACQUITY UPLC I-Class (Waters Corp., Milford, MA, USA) coupled to a XEVO-G2 XS quadrupole time-of-flight (ToF) mass spectrometer (Waters, Manchester, NH, USA) with an electrospray ionization source. The separation of different metabolites was done in a UPLC Ethylene Bridged Hybrid (BEH) C18 column (2.1 × 100 mm, 1.7 µm) using binary gradient elution of solvents A and B. The mobile phase was A: 0.1% formic acid in water; B: 0.1% formic acid in acetonitrile. The mobile phase was delivered at a flow rate of 0.5 mL/min, initially with 1% B, followed by a linear gradient to 15% B over 3 min. Then the percentage of B was increased to 50% within 3 min. Over the next 4 min, the gradient was ramped up to 95% B, and the amount of B was then decreased to 1% in 1.1 min. Over 2 min, the percentage of B returned to initial conditions (1%), until the end of the chromatographic run (13 min). The column temperature was adjusted to 40 °C. The injection volume was 5 µL. 

Data were acquired in positive electrospray ionization (ESI+) mode with the capillary voltage set to 2.0 kV, the cone voltage to 30 eV and the source temperature to 120 °C. The desolvation gas was nitrogen, with a flow rate of 500 L/h and with a temperature of 350 °C. Data were acquired from *m*/*z* 100 to 2000 in Mass Spectrometry (MS^E^) mode in which the collision energy was alternated between low energy (6 eV) and high energy (ramped up from 20 to 40 eV).

As a look mass for accurate mass measurements, leucine enkephalin (200 pg/µL in acetonitrile: water (50:50 *v*/*v*) + 0.1% formic acid) was infused. For calibration, 0.5 mM sodium formate was used. Five pooled samples (QC) were initially injected to equilibrate the column.

### 4.5. Data Acquisition and Statistical Analysis

The raw MSE datasets were acquired in continuum mode and processed within UNIFI 1.8.1 (Waters Corp., Milford, USA). The analysis parameters were as follows: retention time of 0–10 min and peak width of 1–30 s. Data within UNIFI 1.8.1 were passed through the apex peak detection and alignment processing algorithms. The intensity of each ion was normalized with respect to the total ion count (TIC) to generate a data matrix that consisted of the retention time, *m*/*z* value, and the normalized peak area.

The multivariate data matrix was analyzed by EZinfo software (Waters Corp., Milford, MA, USA) and the univariate analysis was performed with MetaboAnalyst [23]. The data were mean-centered and Pareto-scaled prior to principal component analysis (PCA) and orthogonal projection to latent structures discriminate analysis (OPLS-DA). Potential markers of interest were extracted from the combining VIP plot that was constructed from the loading plots of OPLS-DA. A VIP threshold of 1.5 was considered to select the metabolites.

The nonparametric univariate method, Mann–Whitney–Wilcoxon test was applied to measure the significance of each metabolite, with results adjusted for multiple testing using false discovery rate (FDR) correction, with a mass tolerance of 10 ppm.

Exact molecular mass data (*m*/*z*) from significant peaks were used to search the online Human Metabolome Database (HMDB) (http://www.hmdb.ca) for metabolite identities. The identities of key metabolites were confirmed by inspecting the MSE spectra and by comparison of fragmentation pattern with those reported in the HMDB database. 

### 4.6. Pathways Analysis

Metabolite Set Enrichment Analysis and Pathway Analysis were carried out using the pathway analysis module (MetPA) of MetaboAnalyst 3.0. Hypergeometric test and relative betweenness centrality were used for over-representation analysis and pathway topology analysis, respectively.

## 5. Conclusions

In our pilot study conducted with Mexican women in their the 3rd trimester of pregnancy and previously diagnosed with GDM, we identified 14 metabolites belonging to different classes of compounds which suggest biochemical and metabolic changes orchestrated due to GDM physiopathology. This is, to our knowledge, the first metabolomics study conducted in Mexican women diagnosed with GDM. We found that metabolites from steroid hormone biosynthesis and TRP metabolism pathways could have a significant role in GDM and may be associated with different negative outcomes. The upregulation of these pathways, as a consequence of the oxidative stress and inflammation persistent in GDM, could lead to a higher IR, predisposing to several diabetes-associated complications. These metabolites need to be investigated as potential biomarkers for prognostication in future follow-up studies.

## Figures and Tables

**Table 1:** General clinical characteristics of the pregnant women included in the study.

	GDM (*n* = 24)	Control (*n* = 11)	*p*-Value
Age (years)	31.00 ± 1.401	26.91 ± 1.232	0.0771
Gestational age at sampling (weeks)	32.05 ± 0.9026	34.74 ± 0.6853	0.0672
BMI (Kg/m^2^)	30.07 ± 1.268	25.98 ± 1.594	0.0546
Glucose (mg/dL)	92.75 ± 3.174	76.70 ± 1.764	0.0024
SBP (mmHg)	110.4 ± 9.079	108.2 ± 9.816	0.8781
DBP (mmHg)	73.75 ± 9.816	71.82 ± 7.109	0.8161
Creatinine (mg/dL)	0.5262 ± 0.02066	0.5500 ± 0.03643	0.5436
Urea (mg/dL)	13.83 ± 0.9461	16.12 ± 1.734	0.2144
Hb (g/dL)	12.52 ± 0.8055	13.26 ± 1.074	0.1453
Leucocytes (×10^3^)	8.739 ± 1.756	8.000 ± 1.525	0.2339

Data are presented as mean ± standard error of mean (SEM). BMI: body mass index; SBP: systolic blood pressure; DBP: diastolic blood pressure. *p*-values were determined by independent t-test or Mann–Whitney test. Statistically significant: *p* < 0.05.

**Table 2:** Differential metabolites dysregulated in GDM urine.

Class	Compound	HMDB	Molecular Formula	Observed *m*/*z*	RT (min)	*p*-value	FC	Mass Error (mDa)	Adducts
Benzopyrans	**5-carboxy-alpha-chromanol**	HMDB0012798	C_19_H_28_O_4_	338.2322	5.82	2 × 10^−4 a^	4.2	2.0	NH4^+^
Carboxylic acids and derivatives	**1-Methyl N-l-alpha-aspartyl-l-phenylalanate (Aspartame)**	HMDB0001894	C_14_H_18_N_2_O_5_	295.12879	3.32	4.45 × 10^−4a^	5.1	0.2	H^+^
Glycerolipids	DG (24:0/14:1)	HMDB0007792	C_41_H_78_O_5_	668.6179	9.87	6.36 × 10^−4 a^	3.6	−0.9	NH^4+^
Indoles and derivatives	l-Tryptophan	HMDB0000929	C_11_H_12_N_2_O_2_	205.09703	2.90	3.08 × 10^−6 a^	2.4	−0.5	H^+^
Tetrapyrroles and derivatives	l-Urobilinogen	HMDB0004157	C_33_H_48_N_4_O_6_	597.3631	5.94	3.75 × 10^−3 a^	3.2	−1.6	H^+^
Sphingolipids	Cer (d18:0/23:0)	HMDB0011767	C_41_H_83_NO_3_	638.60751	9.92	5.0 × 10^−7 a^	11	0.1	H^+^
	SM (d18:0/22:0)	HMDB0012091	C_45_H_93_N_2_O_6_P	789.685	9.62	4.96 × 10^−6 a^	4.2	0.6	H^+^
Steroid and steroid derivatives	11-oxo-androsterone-glucuronide	HMDB0010338	C_25_H_36_O_9_	503.2247	5.5	5.44 × 10^−4 a^	2.6	−0.5	Na^+^
	cortolone-3-glucuronide	HMDB0010320	C_27_H_42_O_11_	543.2789	5.12	5.80 × 10^−4 a^	2.9	−1.1	H^+^
	tetrahydroaldosterone-3-glucuronide	HMDB0010357	C_27_H_40_O_11_	563.2456	5.09	2.38 × 10^−5 a^	4.6	−0.7	Na^+^
	5-androstene-3b,16b,17a-triol	HMDB0000523	C_19_H_30_O_3_	329.21105	5.09	6.10 × 10^−5 a^	4.4	1.9	Na^+^
	21-deoxycortisol	HMDB0004030	C_21_H_30_O_4_	347.22121	5.26	2.45 × 10^−4 a^	3.7	−0.6	H^+^
	11b, 17a,21-Triydroxypreg-nonolone	HMDB0006760	C_21_H_32_O_5_	365.2317	5.09	2.0 × 10^−3 a^	3.1	−0.4	H^+^
	**cucurbitacin c**	HMDB0034706	C_32_H_48_O	561.3411	5.92	3.00 × 10^−4 a^	3.3	0.1	H^+^

a: significative for false discovery rate (FDR) correction. FC: Fold change. Metabolites in bold are exogenous metabolites.

**Table 3:** Diagnostic criteria for GDM.

Procedure	Glucose Cut Points ^a^		
	Time (h)	mg/dL	mmol/L
Step 1: 50 g	Fasting	≥140 mg/dL	7.8
Step 2: 100 g, 3 h OGTT ^b^	Fasting	≥95 mg/dL	5.3
	1	≥180 mg/dL	10.0
	2	≥155 mg/dL	8.6
	3	≥140 mg/dL	7.8
75 g, 2 h OGTT ^b^	Fasting	≥92 mg/dL	5.1
	1	≥180 mg/dL	10.0
	2	≥153 mg/dL	8.4

a: venous serum or plasma glucose measured at the hospital laboratory. b: Two values meeting or exceeding the cut points are required for diagnosis.

## Supplementary Materials

The following are available online at https://www.mdpi.com/1422-0067/20/5/1186/s1, Figure S1: Principal Component Analysis (PCA) scores plots of urine GDM and controls acquired in positive mode. Table S1: Metabolites differentially expressed (*p* < 0.05) predicted from a multivariate model.

## References

[1] Mirghani Dirar A., Doupis J. (2017). Gestational diabetes from A to Z. World J. Diabetes.

[2] Simjak P., Cinkajzlova A., Anderlova K., Parizek A., Mraz M., Krsek M., Haluzik M. (2018). The role of obesity and adipose tissue dysfunction in gestational diabetes mellitus. J. Endocrinol..

[3] Flores-Padilla L., Solorio-Paez I.C., Melo-Rey M.L., Trejo-Franco J. (2014). Pregnancy and obesity: Risk of developing gestational diabetes in the northern border area of Mexico. Gac. Med. Mex..

[4] Smith H.O., Hilgers R.D., Bedrick E.J., Qualls C.R., Wiggins C.L., Rayburn W.F., Waxman A.G., Stephens N.D., Cole L.W., Swanson M. (2003). Ethnic differences at risk for gestational trophoblastic disease in New Mexico: A 25-year population-based study. Am. J. Obstet. Gynecol..

[5] Brink H.S., van der Lely A.J., van der Linden J. (2016). The potential role of biomarkers in predicting gestational diabetes. Endocr. Connect..

[6] Peng S., Zhang J., Liu L., Zhang X., Huang Q., Alamdar A., Tian M., Shen H. (2015). Newborn meconium and urinary metabolome response to maternal gestational diabetes mellitus: A preliminary case-control study. J. Proteome Res..

[7] Damm P. (2009). Future risk of diabetes in mother and child after gestational diabetes mellitus. Int. J. Gynaecol. Obstet..

[8] Ramirez-Torres M.A. (2013). The importance of gestational diabetes beyond pregnancy. Nutr. Rev..

[9] Wang M., Xia W., Li H., Liu F., Li Y., Sun X., Lu S., Xu S. (2018). Normal pregnancy induced glucose metabolic stress in a longitudinal cohort of healthy women: Novel insights generated from a urine metabolomics study. Medicine.

[10] Mao X., Chen X., Chen C., Zhang H., Law K.P. (2017). Metabolomics in gestational diabetes. Clin. Chim. Acta Int. J. Clin. Chem..

[11] Qiu C., Enquobahrie D.A., Frederick I.O., Sorensen T.K., Fernandez M.A., David R.M., Bralley J.A., Williams M.A. (2014). Early pregnancy urinary biomarkers of fatty acid and carbohydrate metabolism in pregnancies complicated by gestational diabetes. Diabetes Res. Clin. Pract..

[12] Dudzik D., Zorawski M., Skotnicki M., Zarzycki W., Kozlowska G., Bibik-Malinowska K., Vallejo M., Garcia A., Barbas C., Ramos M.P. (2014). Metabolic fingerprint of Gestational Diabetes Mellitus. J. Proteome.

[13] Diaz S.O., Pinto J., Graca G., Duarte I.F., Barros A.S., Galhano E., Pita C., Almeida Mdo C., Goodfellow B.J., Carreira I.M. (2011). Metabolic biomarkers of prenatal disorders: An exploratory NMR metabonomics study of second trimester maternal urine and blood plasma. J. Proteome Res..

[14] Graca G., Goodfellow B.J., Barros A.S., Diaz S., Duarte I.F., Spagou K., Veselkov K., Want E.J., Lindon J.C., Carreira I.M. (2012). UPLC-MS metabolic profiling of second trimester amniotic fluid and maternal urine and comparison with NMR spectral profiling for the identification of pregnancy disorder biomarkers. Mol. Biosyst..

[15] Sachse D., Sletner L., Morkrid K., Jenum A.K., Birkeland K.I., Rise F., Piehler A.P., Berg J.P. (2012). Metabolic changes in urine during and after pregnancy in a large, multiethnic population-based cohort study of gestational diabetes. PLoS ONE.

[16] Law K.P., Han T.L., Mao X., Zhang H. (2017). Tryptophan and purine metabolites are consistently upregulated in the urinary metabolome of patients diagnosed with gestational diabetes mellitus throughout pregnancy: A longitudinal metabolomics study of Chinese pregnant women part 2. Clin. Chim. Acta Int. J. Clin. Chem..

[17] Debnath S., Velagapudi C., Redus L., Thameem F., Kasinath B., Hura C.E., Lorenzo C., Abboud H.E., O’Connor J.C. (2017). Tryptophan Metabolism in Patients With Chronic Kidney Disease Secondary to Type 2 Diabetes: Relationship to Inflammatory Markers. Int. J. Tryptophan Res. IJTR.

[18] Oxenkrug G. (2013). Insulin resistance and dysregulation of tryptophan-kynurenine and kynurenine-nicotinamide adenine dinucleotide metabolic pathways. Mol. Neurobiol..

[19] Bentley-Lewis R., Xiong G., Lee H., Yang A., Huynh J., Kim C. (2014). Metabolomic analysis reveals amino acid responses to an oral glucose tolerance test in women with prior history of gestational diabetes mellitus. J. Clin. Transl. Endocrinol..

[20] Bentley-Lewis R., Huynh J., Xiong G., Lee H., Wenger J., Clish C., Nathan D., Thadhani R., Gerszten R. (2015). Metabolomic profiling in the prediction of gestational diabetes mellitus. Diabetologia.

[21] Marvan M.L., Islas M., Vela L., Chrisler J.C., Warren E.A. (2008). Stereotypes of women in different stages of their reproductive life: Data from Mexico and the United States. Health Care Women Int..

[22] Mistry H.D., Eisele N., Escher G., Dick B., Surbek D., Delles C., Currie G., Schlembach D., Mohaupt M.G., Gennari-Moser C. (2015). Gestation-specific reference intervals for comprehensive spot urinary steroid hormone metabolite analysis in normal singleton pregnancy and 6 weeks postpartum. Reprod. Biol. Endocrinol. RB&E.

[23] Gueuvoghlanian-Silva B.Y., Cordeiro F.B., Lobo T.F., Cataldi T.R., Lo Turco E.G., Bertolla R.P., Mattar R., Torloni M.R., Daher S. (2015). Lipid fingerprinting in mild versus severe forms of gestational diabetes mellitus. PLoS One.

[24] Mizugishi K., Inoue T., Hatayama H., Bielawski J., Pierce J.S., Sato Y., Takaori-Kondo A., Konishi I., Yamashita K. (2015). Sphingolipid pathway regulates innate immune responses at the fetomaternal interface during pregnancy. J. Biol. Chem..

[25] O’Neill K., Alexander J., Azuma R., Xiao R., Snyder N.W., Mesaros C.A., Blair I.A., Pinney S.E. (2018). Gestational Diabetes Alters the Metabolomic Profile in 2nd Trimester Amniotic Fluid in a Sex-Specific Manner. Int. J. Mol. Sci..

[26] Lappas M., Mundra P.A., Wong G., Huynh K., Jinks D., Georgiou H.M., Permezel M., Meikle P.J. (2015). The prediction of type 2 diabetes in women with previous gestational diabetes mellitus using lipidomics. Diabetologia.

[27] Dong Y., Chen Y.T., Yang Y.X., Shou D., Li C.Y. (2016). Urinary Metabolomic Profiling in Zucker Diabetic Fatty Rats with Type 2 Diabetes Mellitus Treated with Glimepiride, Metformin, and Their Combination. Molecules.

[28] Huhtala M.S., Tertti K., Pellonpera O., Ronnemaa T. (2018). Amino acid profile in women with gestational diabetes mellitus treated with metformin or insulin. Diabetes Res. Clin. Prac..

[29] Zawiejska A., Wender-Ozegowska E., Grewling-Szmit K., Brazert M., Brazert J. (2016). Short-term antidiabetic treatment with insulin or metformin has a similar impact on the components of metabolic syndrome in women with gestational diabetes mellitus requiring antidiabetic agents: Results of a prospective, randomised study. J. Physiol. Pharmacol..

[30] Barrett H.L., Dekker Nitert M., Jones L., O’Rourke P., Lust K., Gatford K.L., De Blasio M.J., Coat S., Owens J.A., Hague W.M. (2013). Determinants of maternal triglycerides in women with gestational diabetes mellitus in the Metformin in Gestational Diabetes (MiG) study. Diabetes Care.

[31] Barrett H.L., Gatford K.L., Houda C.M., De Blasio M.J., McIntyre H.D., Callaway L.K., Dekker Nitert M., Coat S., Owens J.A., Hague W.M. (2013). Maternal and neonatal circulating markers of metabolic and cardiovascular risk in the metformin in gestational diabetes (MiG) trial: Responses to maternal metformin versus insulin treatment. Diabetes Care.

[32] Chiswick C., Reynolds R.M., Denison F., Drake A.J., Forbes S., Newby D.E., Walker B.R., Quenby S., Wray S., Weeks A. (2015). Effect of metformin on maternal and fetal outcomes in obese pregnant women (EMPOWaR): A randomised, double-blind, placebo-controlled trial. Lancet Diabetes Endocrinol..

[33] McCarthy E.A., Walker S.P., Ugoni A., Lappas M., Leong O., Shub A. (2016). Self-weighing and simple dietary advice for overweight and obese pregnant women to reduce obstetric complications without impact on quality of life: A randomised controlled trial. BJOG Int. J. Obstet. Gynaecol..

[34] Vejrazkova D., Vcelak J., Vankova M., Lukasova P., Bradnova O., Halkova T., Kancheva R., Bendlova B. (2014). Steroids and insulin resistance in pregnancy. J. Steroid Biochem. Mol. Biol..

[35] Chen T., Zheng X., Ma X., Bao Y., Ni Y., Hu C., Rajani C., Huang F., Zhao A., Jia W. (2016). Tryptophan Predicts the Risk for Future Type 2 Diabetes. PLoS ONE.

[36] Yu E., Papandreou C., Ruiz-Canela M., Guasch-Ferre M., Clish C.B., Dennis C., Liang L., Corella D., Fito M., Razquin C. (2018). Association of Tryptophan Metabolites with Incident Type 2 Diabetes in the PREDIMED Trial: A Case-Cohort Study. Clin. Chem..

[37] Leitner M., Fragner L., Danner S., Holeschofsky N., Leitner K., Tischler S., Doerfler H., Bachmann G., Sun X., Jaeger W. (2017). Combined Metabolomic Analysis of Plasma and Urine Reveals AHBA, Tryptophan and Serotonin Metabolism as Potential Risk Factors in Gestational Diabetes Mellitus (GDM). Front. Mol. Biosci..

[38] Badawy A.A., Namboodiri A.M., Moffett J.R. (2016). The end of the road for the tryptophan depletion concept in pregnancy and infection. Clin. Sci..

[39] von Bubnoff D., Matz H., Frahnert C., Rao M.L., Hanau D., de la Salle H., Bieber T. (2002). FcepsilonRI induces the tryptophan degradation pathway involved in regulating T cell responses. J. Immunol..

[40] Committee on Practice B.-O. (2018). ACOG Practice Bulletin No. 190: Gestational Diabetes Mellitus. Obstet. Gynecol..

[41] Huhn E.A., Massaro N., Streckeisen S., Manegold-Brauer G., Schoetzau A., Schulzke S.M., Winzeler B., Hoesli I., Lapaire O. (2017). Fourfold increase in prevalence of gestational diabetes mellitus after adoption of the new International Association of Diabetes and Pregnancy Study Groups (IADPSG) criteria. J. Perinat. Med..

