# Supplementary material for: Urinary Metabolites Altered during the Third Trimester in Pregnancies Complicated by Gestational Diabetes Mellitus: Relationship with Potential Upcoming Metabolic Disorders
Source: Int J Mol Sci. 2019 Mar 8;20(5):1186. doi: 10.3390/ijms20051186 (PMC6429483; doi:10.3390/ijms20051186)
Supplement: Supplementary file 1 [file ijms-20-01186-s001.zip › Supplementary Finals Figures/Supplementary Table S1 ijms.docx]

Supplementary Table S1: Differentially expressed metabolites (p < 0.05) between GDM and controls

| **CLASS** | **Compound** | **HMDB** | **Molecular Formula** | **Observed m/z** | **RT**  **(min)** | **p value** | **FC** | **Mass error (mDa)** | **Adducts** |
| --- | --- | --- | --- | --- | --- | --- | --- | --- | --- |
| Benzimidazoles | **(S) esomeprazole** | HMDB0005009 | C_17_H_19_N_3_O_3_S | 346.1239 | 0.91 | 1 x 10^-2^ | 1.9 | 1.9 | H+ |
| Benzopyrans | **7'-Carboxy-alpha-tocotrienol** | HMDB0012849 | C_21_H_30_O_4_ | 347.2214 | 5.09 | 2.7 x 10^-3^ | 3.1 | 0.3 | H+ |
| Carboxylic acids | Creatinina | HMDB0000562 | C_4_H_7_N_3_O | 114.06625 | 0.54 | 4.27 x 10^-4^ | 1.6 | -0.2 | H+ |
|  | Asymmetric dimethylarginine | HMDB0001539 | C_8_H_18_N_4_O_2_ | 203.15023 | 0.56 | 2.49 x 10^-2^ | 1.7 | -0.2 | H+ |
|  | Leucylproline | HMDB0011175 | C_11_H_20_N_2_O_3_ | 229.15462 | 1.91 | 6.58 x 10^-3^ | 4.9 | -0.3 | H+ |
|  | Pentosidine | HMDB0003933 | C_17_H_33_NO_4_ | 379.2111 | 5.23 | 4.0 x 10^-3^ | 3.0 | 2.3 | H+ |
|  | D-threo-Isocitric acid | HMDB0001874 | C_6_H_8_O_7_ | 215.0162 | 0.78 | 2.99 x 10^-2^ | 1.5 | -0.1 | Na+ |
| Fatty Acyls | 3-Methylglutarylcarnitine | HMDB0000552 | C_13_H_23_NO_6_ | 290.15976 | 2.59 | 1.09 x 10^-2^ | 2.4 | -0.3 | H+ |
|  | Butyrylcarnitine | HMDB0002013 | C_11_H_21_NO_4_ | 232.15433 | 2.70 | 4.57 x 10^-2^ | 1.9 | -0.2 | H+ |
|  | L-Octanoylcarnitine | HMDB0000791 | C_15_H_29_NO_4_ | 310.20123 | 5.4 | 3.65 x 10^-2^ | 1.8 | 2.3 | Na+ |
|  | 2-Methylbutyroylcarnitine | HMDB0000378 | C_12_H_23_NO_4_ | 246.1699 | 3.46 | 3.43 x 10^-2^ | 2.2 | -0.1 | NH4+ |
|  | Tridecanoic acid | HMDB0000910 | C_13_H_26_O_2_ | 253.1588 | 4.61 | 4.4 x 10^-2^ | 1.9 | 3.4 | K+ |
| Glycerophospholipids | Phosphatidyl-D-myo-inositol | HMDB0006953 | C_11_H_19_O_13_P | 413.0424 | 3.45 | 3.7 x 10^-3^ | *1.5 | -3.2 | Na+ |
| Hydroxy acids and derivatives | 9-Decenoylcarnitine | HMDB0013205 | C_17_H_31_NO_4_ | 314.23197 | 5.66 | 7.12 x 10^-3^ | 6 | -0.6 | H+ |
|  | 3, 5-Tetradecadiencarnitine | HMDB0013331 | C_21_H_37_NO_4_ | 385.30941 | 5.92 | 6.09 x 10^-3^ | 2.7 | 3.3 | NH4+ |
|  | 6-Keto-decanoylcarnitine | HMDB0013202 | C_17_H_31_NO_5_ | 330.2269 | 5.12 | 2 x 10^-3^ | 1.9 | -0.6 | H+ |
| Imidazopyrimidines | Uric Acid | HMDB0000289 | C_5_H_4_N_4_O_3_ | 169.03576 | 0.76 | 3.45 x 10^-2^ | 1.3 | -0.1 | H+ |
|  | 7-Methylguanine | HMDB0000897 | C_6_H_7_N_5_O | 166.07234 | 0.87 | 2.48 x 10^-2^ | 1.6 | -0.1 | H+ |
|  | 1-Methylhypoxanthine | HMDB0013141 | C_6_H_6_N_4_O | 151.06137 | 2.32 | 1.00 x 10^-2^ | 2 | 0 | H+ |
| Indoles and derivatives |  |  |  |  |  |  |  |  |  |
|  | Indoleacrylic acid | HMDB0000734 | C_11_H_9_NO_2_ | 188.07074 | 2.90 | 9.12 x 10^-3^ | 1.8 | -0.1 | H,NH4+ |
| Organonitrogen compounds | **1,1-Dimethylbiguanide** | HMDB0001921 | C_4_H_11_N_5_ | 130.10879 | 0.60 | 4.52 x 10^-2^ | 1.5 | -0.1 | H+ |
| Organooxygen compounds | 1-(beta-D-Ribofuranosyl)-1,4-dihydronicotinamide | HMDB0011648 | C_11_H_16_N_2_O_5_ | 257.11309 | 1.89 | 1.07 x 10^-2^ | 3.3 | -0.3 | H+ |
|  | Acetyl-N-formyl-5-methoxykynurenamine | HMDB0004259 | C_13_H_16_N_2_O_4_ | 265.1181 | 3.56 | 8.75 x 10^-3^ | 1.4 | -0.5 | H+ |
| Prenol Lipids | 11'-Carboxy-alpha-tocotrienol | HMDB0012516 | C_26_H_38_O_4_ | 432.3160 | 6.02 | 1.07 x 10^-3^ | 7.0 | -0.8 | NH4+ |
| Purine nucleosides | 1-Methyladenosine | HMDB0003331 | C_11_H_15_N_5_O_4_ | 282.11961 | 0.88 | 1.65 x 10^-2^ | 1.5 | -0.3 | H+ |
|  | Succinyladenosine | HMDB0000912 | C_14_H_17_N_5_O_8_ | 384.11463 | 2.60 | 1.64 x 10^-2^ | 1.9 | -0.4 | H+ |
|  | N2,N2-Dimethylguanosine | HMDB0004824 | C_12_H_17_N_5_O_5_ | 312.1304 | 2.64 | 4.4 x 10^-3^ | 2.0 | 0.1 | H+ |
| Pyridines and derivatives | N1-Methyl-4-pyridone-3-carboxamide | HMDB0004194 | C_7_H_8_N_2_O_2_ | 153.06584 | 1.52 | 2.71 x 10^-2^ | 1.6 | -0.2 | H+ |
| Pyrrolopyrimidines | 7-Aminomethyl-7-carbaguanine | HMDB0011690 | C_7_H_9_N_5_O | 180.08755 | 2.64 | 1.42 x 10^-2^ | 1.9 | 0 | H+ |
| Steroid hormones and derivatives | Estrone | HMDB0000145 | C_18_H_22_O_2_ | 271.16924 | 4.60 | 3.89 x 10^-2^ | 1.8 | -0.4 | H+ |
|  | 18-Hydroxycortisol | HMDB0000418 | C_21_H_30_O_6_ | 379.21088 | 5.22 | 4.00 x 10^-3^ | 3 | 1.7 | H+ |
|  | Glycochenodeoxycholic acid 3-glucuronide | HMDB0002579 | C_32_H_51_NO_11_ | 626.35317 | 6.05 | 2.69 x 10^-2^ | 4.1 | 0.2 | H+ |
|  | 5alpha-Androst-16-en-3-one | HMDB0034406 | C_19_H_28_O | 273.22105 | 6.18 | 2.04 x 10^-2^ | 2.4 | -0.3 | H+ |
|  | 3a,5b,7a,12a)-24-(carboxymethyl)amino-1,12-dihydroxy-24-oxocholan-3-yl-b-D-Glucopyranosiduronic acid | HMDB0002472 | C_32_H_51_NO_12_ | 642.348 | 4.82 | 9.41 x 10^-3^ | 12.1 | -0.1 | H+ |
|  | 11-Hydroxyprogesterone 11-glucuronide | HMDB0010364 | C_27_H_38_O_9_ | 507.2585 | 5.12 | 7.53 x 10^-3^ | 3.7 | -0.4 | H+ |
|  | Estriol-16-Glucuronide | HMDB0006766 | C_24_H_32_O_9_ | 487.1911 | 4.6 | 3.0 x 10^-2^ | 1.6 | -2.8 | Na+ |
|  | Androstenedione | HMDB0000053 | C_19_H_26_O_2_ | 287.2010 | 4.93 | 4.7 x 10^-2^ | 16.4 | 0.4 | H+ |
|  | Pregnanediol-3-glucuronide | HMDB0010318 | C_27_H_44_O_8_ | 519.2923 | 6.4 | 2.7 x 10^-2^ | 2.4 | -0.5 | Na+ |
