# Supplementary figures and images for: Urinary Metabolites Altered during the Third Trimester in Pregnancies Complicated by Gestational Diabetes Mellitus: Relationship with Potential Upcoming Metabolic Disorders
Source: Int J Mol Sci. 2019 Mar 8;20(5):1186. doi: 10.3390/ijms20051186 (PMC6429483; doi:10.3390/ijms20051186)

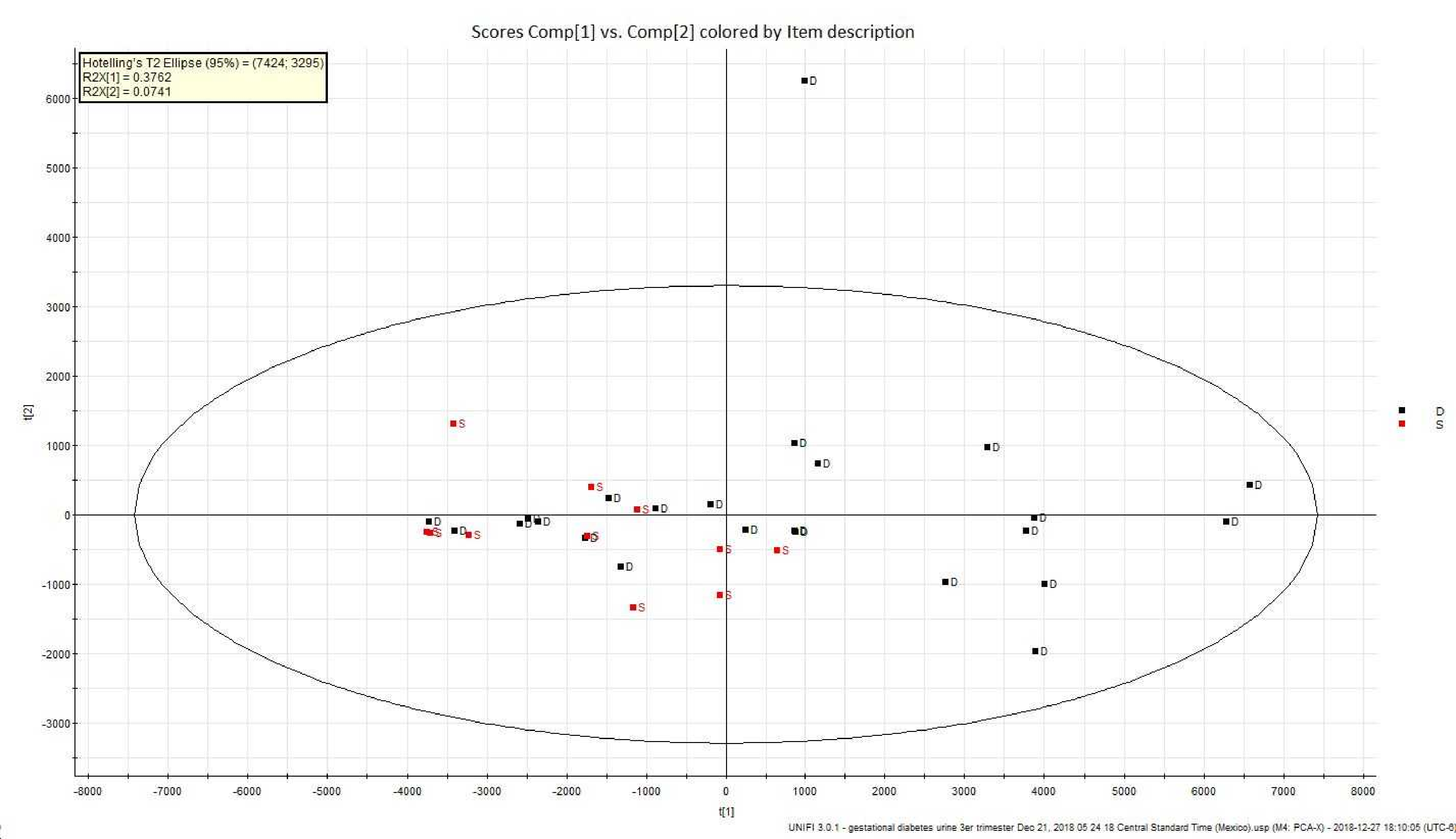

Supplement: Supplementary file 1 [file ijms-20-01186-s001.zip › Supplementary Finals Figures/SUPPLEMENTARY FIGURE 1_001.tif]
